# Supplementary material for: Patterns of Intron Gain and Loss in Fungi
Source: PLoS Biol. 2004 Nov 30;2(12):e422. doi: 10.1371/journal.pbio.0020422 (PMC532390; doi:10.1371/journal.pbio.0020422)
Supplement: Table S1 — Also available at http://genes.mit.edu/NielsenEtAl/. (4.3 MB ZIP). [file pbio.0020422.st001.zip › NielsenEtAl/html/721.html]

AN4259.1.NCU02813.1.MG01595.1.FG07122.1


```
 CLUSTAL W (1.82) Multiple Sequence Alignments - Introns Inserted


Sequence 1: NCU02813.1	434 aa
Sequence 2: MG01595.1	432 aa
Sequence 3: FG07122.1	446 aa
Sequence 4: AN4259.1	459 aa
Alignment Length: 483 aa
Number Identitical Residues: 109 aa
Alignment Score (without introns) 7091


MG01595.1 	---MTASYQPQLVFVDGSFAELAQDMASVL-------------QISDEIQPLLDSEKE--
NCU02813.1	---MAT---PQLIFVDGTFAELAQEMADFV-------------QVGEQVQPLLEQDKR--
FG07122.1 	MADTKTAGQPTLVFVDGSFEDLAAEMAEYL-------------KAEDAKQ-LLSQEKQP-
AN4259.1  	---MPAP--STTLLIEGSFSELAEEFAAYLDALNKPDDTTVQTEVAPLLQPLREQEQNDT
          	     :.  .  ::::*:* :** ::*  :.: .....:: .::     *.* ..::. :

MG01595.1 	-------SEALTKIVSESKKLNAIPEKE~FTGAYNLLVHLVLQSKEPKKHLPTICQNLTR
NCU02813.1	-------EDALDLIVKASHSLNAAPEKD~FTPAYNLLIHLVLQSEEPKKYLPTLCANLLK
FG07122.1 	-----SNEEVLSKLVSASGALNTVPEKE~YTAASNLMTHLVLQSSDPKKFLPTLCTTFAK
AN4259.1  	QLDQSKRDEVLKKLVSAATVLNTAPEKE1ITPAYNLLIHLVQQASDPDMFLSRICSYLAK
          	. ..:. .:.*  :*. :  **: ***:  * * **: *** *:.:*. .*. :*  : :

MG01595.1 	PVTSSPQHGAQLALFELTSIFNLLKPNDPVRFNVFIQIIRFYKI-HSIPISDHLKSALK-
NCU02813.1	PITSSPTHGFTLASNALTTIFNLLDKSNPLRFNVFMQIVRFIRQ-NSQ--FDLLKPRLK-
FG07122.1 	PLVNSPVHGVGLSLNALTTVFNLLEPSNPVRARVFMEILKFLRS-HAM--FESLRTYLD-
AN4259.1  	PIPSSPQFGASLSIAILSTIFNTLAPTDSSRFHVLLAIVTVIRQSGSSAAFDALKPQLTA
          	*: .** .*  *:   *:::** *  .:. * .*:: *: . : : : .  : *:. * :

MG01595.1 	QLPRWLQSWELDEEDQRKMYSEVIEVMTAAGEEE2EAYQHILKALRTFDSEDAEDYTSEE
NCU02813.1	NVEGWLQEWKSDSESQRKLYVEVSDAANDGGDQE2ESYFYLLKALATFDRDNQDEVASDE
FG07122.1 	KLPEWLTTWGTDADFQRKIYEEVAEVALEAGEEQ2QAYEYVVKALRTFDG---DAISSED
AN4259.1  	QLPNWRSTWELDDEEARRLHLAIADAAQAAGDLD~WAQTHVVDALQTIPP---AEASSPA
          	::  *   *  * :  *:::  : :.   .*: :  :  :::.** *:        :*  

MG01595.1 	AQQLALRALRSAISSPTRLSFEDIRALPAVHALSESHPVHYQLLQIFGEQDLDDYDDFRE
NCU02813.1	AQKLSLRALRMAISSPTRFDFNDLRSLPSVHALSDSHPVYSQLLDIFTEQDLEDYNDFRD
FG07122.1 	AQRLSLRAVKKALTSSNHFLFQDLRGIPSVQALSDSHPVYSQLLDIFAEQDLEDYNDFND
AN4259.1  	ARDLAVRALTSALTHPAVFDFTPLTAADAVQALRSSDAPLFELLEIFTADTLDAYEDFIS
          	*: *::**:  *:: .  : *  : .  :*:** .*..   :**:**  : *: *:** .

MG01595.1 	EH--EGFIEKENLD--NEVLYRKMRLLTFASLAAAS-----MQTREISYNSITKALQIPS
NCU02813.1	EH--QGWIENEKLD--HEKLQRKMRLLTFASLAAS------TPNREIPYASIAKALQIPD
FG07122.1 	EH--QGWVEKESLD--HEKLHRKMRLLTFSSLAAS------TPSREIEYSKITKALQIPE
AN4259.1  	ATPVENILPDNALAPHAEALQTKIRLLTLASLAAAATTTTGPSARSLSYETIASALRVPQ
          	  . :. : .: * .  * *  *:****::****::::::.   *.: * .*:.**::*.

MG01595.1 	EDVEMWAIDVIRAGLVEGKLSQKKKVFLIHSVRYRVFGEKQWRQLASSLEKTKKTVSTLL
NCU02813.1	EDVEMWTIDVIRSKLVEGRLSQKQKVFLVHRTTYRVFGEKQWRELGTRVDQFKTVVDRLV
FG07122.1 	DEIEMWAIDVIRAGLVEGKLSQQRQKFLVHKVTYRVFGQKQYQELANRVDHWRTTLQNVL
AN4259.1  	EEVEKWVIDTIRAGLVEGKLSQLRSEFLVHRATYRVFGEKQWAEVQGRLMVWRRSLENVL
          	:::* *.**.**: ****:*** :. **:* . *****:**: ::   :   :  :. ::

MG01595.1 	QTLRREEAN-------AQQEAERKLVEASTQHNNDRGNQRRGGN-------RGQ-QHRER
NCU02813.1	GVVRKAQTDVEAQREREQQELERKLANAGLGESEQRGGNRGGRT-------GGARQTRQR
FG07122.1 	GVLRQEQANAKSQKEREIQELERKVANVGMGGAQDRRRQNQNREPREPREPREPREPKER
AN4259.1  	GVIRSERER-------FVREAAAAAAAAAAEGEKGDKNNKGPSE--------RRRAPQEI
          	 .:*  .           :*     . ..    :    :.                 :: 

MG01595.1 	NDNDD
NCU02813.1	TDDDD
FG07122.1 	TEDDD
AN4259.1  	AAAE-
          	   :
```
